# The Genetic Variant on Chromosome 10p14 Is Associated with Risk of Colorectal Cancer: Results from a Case-Control Study and a Meta-Analysis

**DOI:** 10.1371/journal.pone.0064310

**Published:** 2013-05-22

**Authors:** Qin Qin, Li Liu, Rong Zhong, Li Zou, Jieyun Yin, BeiBei Zhu, BeiBei Cao, Wei Chen, Jigui Chen, Xiaorong Li, Tingting Li, Xuzai Lu, Jiao Lou, Juntao Ke, Sheng Wei, Xiaoping Miao, Shaofa Nie

**Affiliations:** 1 Department of Epidemiology and Biostatistics, and the Ministry of Education Key Lab of Environment and Health, School of Public Health, Tongji Medical College, Huazhong University of Science and Technology, Wuhan, Hubei, China; 2 Department of Surgery, The Eighth Hospital of Wuhan, Wuhan, Hubei, China; The Children's Hospital of Philadelphia, United States of America

## Abstract

**Background:**

A common single nucleotide polymorphism (SNP), rs10795668, located at 10p14, was first identified to be significantly associated with risk of colorectal cancer (CRC) by a genome-wide association study (GWAS) in 2008; however, another GWAS and following replication studies yielded conflicting results.

**Methods:**

We conducted a case-control study of 470 cases and 475 controls in a Chinese population and then performed a meta-analysis, integrating the current study and 9 publications to evaluate the association between rs10795668 and CRC risk. Heterogeneity among studies and publication bias were assessed by the *χ^2^*-based Q statistic test and Egger's test, respectively.

**Results:**

In the case-control study, significant association between the SNP and CRC risk was observed, with per-A-allele OR of 0.71 (95%CI: 0.54–0.94, *P* = 0.017). The following meta-analysis further confirmed the significant association, with per-A-allele OR of 0.91 (95%CI: 0.89–0.93, *P_heterogeneity_*>0.05) in European population and 0.86 (95%CI: 0.78–0.96, *P*_heterogeneity_ <0.05) in Asian population. Besides, sensitivity analyses and publication bias assessment indicated the robust stability and reliability of the results.

**Conclusions:**

Results from our case-control study and the followed meta-analysis confirmed the significant association of rs10795668 with CRC risk.

## Introduction

Colorectal cancer(CRC) is the third most commonly cancer in males and the second in females, with over 1.2 million new cases and 608,700 deaths in 2008 worldwide[1]. In developed countries, according to the Surveillance Epidemiology and End Results database, CRC is the second leading cause of cancer death in developed countries, with the lifetime risk estimated to be 5–6%[2]. In developing countries, such as China, the incidence rate of CRC have grown rapidly in the past decades, especially in urban areas[3]. Shanghai, as a developed city in China, experienced an annual increase of 4.2% in colorectal cancer incidence, which was even higher than the global level (2%)[4].

CRC is provoked by interactions between genetic and environmental factors. Among all causes for CRC, inherited genetic factors account for approximately 35% of the disease etiology [5]. However, high-penetrance mutations, such as those in *APC*, *SMAD4* and *MMR* genes, are responsible for less than 5% of cases in the pathogenesis of CRC[6]. Mildly or moderately penetrant alleles could explain about 8.3% of etiology in cases with familial aggregation [7], [8]. It is expected that the remaining proportion of inherited susceptibility is likely to be explained by low-risk variants[6].

Genome-wide association studies (GWAS), which made possible the genotyping of hundreds of thousands of single nucleotide polymorphisms (SNPs) that tag linkage disequilibrium (LD) blocks in the genome, have successfully identified novel susceptibility loci for CRC[9]–[19]. Among these loci, the SNP, rs10795668, located at 10p14, was firstly identified by Tomlinson, IP et al. to be significantly associated with CRC risk in a 4-phase GWAS with 18831 cases and 18540 controls in Europeans[18]. However, another GWAS reported by Peters, U. et al. found no role of this variant in CRC susceptibility by a test set of 2906 cases and 3416 controls and 10 replication sets of 8161 cases and 9101 controls in Europeans[17]. In replication studies, some provided statistical evidence of this SNP for CRC risk [20]–[22], however, much more failed to replicate this association [6], [23]–[28]. Besides, rs10795668 exhibited a population difference among the European, Japanese and African American populations [23]. Since allelic frequency and LD patterns across different populations are different, this variant has to be replicated to ascertain its role in CRC development. The replication studies of the association between rs10795668 polymorphism and CRC have been conducted in Northern, Southern and Hong Kong Chinese [20], [22], [25]. Herein, we performed a replication study comprising 470 cases and 475 controls in Central Chinese. Moreover, a meta-analysis combining the current study and previously published studies about rs10795668 was further conducted to clarify the relationship between this SNP and CRC risk.

## Materials and Methods

### Ethics statement

The study was approved by the institutional review board of Tongji Medical College of Huazhong University of Science and Technology. And all participants provided written informed consent to participate in this study.

### Study populations

The study population consisted of 470 CRC cases with newly diagnosed CRC and 475 cancer-free controls. Patients were consecutively recruited between January 1, 2007 and November 31, 2009 at the Eighth Hospital of Wuhan, central China, which is a hospital specializing in colorectal diseases and absorbing majority of colorectal patients in Wuhan and nearby regions. Controls were cancer-free individuals living in Wuhan city and surrounding regions, who were selected from a pool of healthy volunteers visiting the health check-up center at the same hospital during the same period of the recruitment of CRC patients. The inclusion criteria for patients included histopathologically confirmed CRC, without previous chemotherapy or radiotherapy and no restriction in regards to sex, age or disease stage. Cases with pathology report designated familial adenomatous polyposis, inflammatory bowel disease, colorectal adenoma, Lynch syndrome, intestinal tuberculosis and schistosomiasis were not eligible. The selection criteria for controls included cancer-free individuals and frequency matched to cases by sex and age (±5 years). All subjects were unrelated ethnic Han Chinese. Demographic and lifestyle information, and medical data were collected by trained interviewers via direct interview using standardized questionnaires. Clinical data were abstracted from hospital medical records. 5-ml peripheral venous blood was drawn from each participant.

### Genotyping

Genomic DNA was extracted from peripheral blood sample using the RelaxGene Blood System DP319-02 (Tiangen, Beijing, China) according to the manufacturer's instructions. The rs10795668 SNP was genotyped by using the Sequenom MassARRAY platform (Sequenom San diego, CA, USA). Genotypes were called using MassARRAY Typer 4.0 software [29], [30]. To ensure quality control, 5% duplicated samples were randomly selected to assess the reproducibility, with a concordance rate of 100%.

### Statistical analysis

Hardy–Weinberg equilibrium (HWE) was tested by a goodness-of-fit *χ^2^* test to compare the observed genotype frequencies to the expected genotype frequencies in controls. Pearson's *χ^2^* test was used to compare the differences in distribution of categorical variables (sex, smoking status, alcohol use and family history of cancer) and Student's *t*-test was used for continuous variables [age and body weight index (BMI)]. For the SNP with minor allele frequency (MAF) of 0.35, the power of our sample size was calculated to be 0.72 to detect an OR of 0.7 in case-control study.

For the main effect of rs10795668, unconditional Logistical Regression was conducted to calculate odds ratios (ORs) and their corresponding 95% confidence intervals (CIs), adjusted for potential confounders (age, sex, smoking status, alcohol use and BMI). ORs and 95% CIs as the metrics of effect size were calculated for genotypes AG versus GG and AA versus GG. In order to avoid the assumption of genetic models, dominant, recessive and additive models were also analyzed. Two-factor gene-environment interaction analyses were evaluated by using likelihood ratio tests comparing nested models with only main effects and models with main effects plus the relevant interaction term. There environmental factors, such as smoking status, alcohol use and BMI, were included in the interaction models. According to the WHO Expert Consultation's conclusion, the BMI cut-off point for overweight for Chinese was set to be 24 kg/m^2^ [31]. The Bonferroni method was applied for multiple comparison correction. All statistical analyses were performed using SPSS software 12.0 (SPSS, Inc., Chicago, III) and all *P* values were two-tailed tested with a significant level at 0.05.

### Meta-analysis of rs10795668 in association with CRC risk

To further investigate the association between rs10795668 and CRC risk, a meta-analysis was conducted in accordance with the guidelines of the Preferred Reporting Items for Systematic Reviews and Meta-analyses (PRISMA) statement (http://www.prisma-statement.org) (**Checklist S1**).

A systematic literature searching was performed in PubMed/MEDLINE and ISI Web of Science without language restriction up to the end of November, 2012. The search strategy was based on combinations of the terms “rs10795668 or 10p14” and “colorectal cancer or colorectal neoplasm”. To expand the coverage of our searches, we further performed searches in Chinese Biomedical database(CBM) based on the same strategy[32]. In addition, references in retrieved articles were scanned. Reviews, comments, and letters were also checked for additional studies. Searching was performed in duplicate by two independent reviewers (Qin Qin and Li Liu).

Studies were included if they met the all of the following criteria: (1) original study; (2) assessment of the association between rs10795668 and CRC risk; (3) case-control or cohort study design; (4) providing odds ratio (OR) with corresponding confidence 95% interval (95%CI) or sufficient data to calculate them; (5) studies of humans; (6) the genotypes of rs10795668 in controls are in HWE. We left out investigations in subjects with Lynch syndrome. Case reports, comments, reviews and editorials were also excluded. If the studies had overlapping subjects, only the study with the largest population was finally included. If more than one geographic or ethnic population were included in one report, each population was considered separately. All data were extracted independently by two reviewers (Qin Qin and Li Liu) and any disagreement was adjudicated by a third author (Rong Zhong).

The following information was extracted from each study: first author's name, publication year, study country, ethnicity of study population, study design, study type, genotyping method, numbers of cases and controls, control source, MAF. HWE in controls was estimated again in the meta-analysis by the goodness-of-fit *χ^2^* test (*P*<0.01)[33]–[35]. Pooled frequency of A allele of rs10795668 in different ethnic groups was estimated using the inverse variance method described by Thakkinstian A et al.[36]. Statistical heterogeneity across studies included in the meta-analysis was assessed by Cochran's *Q*-statistic and considered significant at *P*<0.05[37]. The *I^2^* statistic was then utilized to estimate heterogeneity quantitatively (*I^2^* = 0–25%, no heterogeneity; *I^2^* = 25–50%, moderate heterogeneity; *I^2^* = 50–75%, large heterogeneity; *I^2^* = 75–100%, extreme heterogeneity)[38]. The fixed-effects model, by inverse variance method, was used to calculate the pooled estimate when heterogeneity was negligible; otherwise, the random-effects model, by DerSimonian and Laird method was applied [39]. Sensitivity analysis was performed to assess the influence of each study on overall estimate by sequential removing of each study[40]. Publication bias was estimated by funnel plot and Eegger's test [41], [42]. All statistical analyses were carried out in STATA statistical software (version11.0; Stata Corporation, college Station, Texas) and all *P* values were two-tailed tested with a significant level at 0.05.

## Results

### Results of Case-control Study

#### Population characteristics

The characteristics of the study subjects were detailed in **Table 1**. There were no significant differences in the distribution of age, sex and alcohol use between cases and controls. The mean age [± standard deviation (SD)] was 58.1(±11.6) years old for cases and 58.4(±13.1) years old for controls (*P* = 0.652). More smokers were observed in cases than in controls (*P*<0.001). In addition, BMI in cases was significantly higher than in controls (*P* = 0.023).

**Table 1:** Characteristics of the study population.

	Cases	Controls	
	No. (%)	No. (%)	*P*
Total	470	475	
Age(years)			0.652^a^
(Mean ± SD)	58.1±11.6	58.4±13.1	
Sex			0.637^b^
Male	258(54.9)	268(56.4)	
Female	212(45.1)	207(43.6)	
Smoking status			8.000×10^−8b^
Never	292(62.1)	371(78.1)	
Ever	178(37.9)	104(21.9)	
Alcohol use			0.073^b^
Never	309(65.7)	338(71.2)	
Ever	161(34.3)	137(28.8)	
BMI			0.023^a^
(Mean ± SD)	23.23±3.41	22.67±3.17	

a Caculated by Student's *t*-test;

b Caculated by Pearson's *χ^2^* test.

#### Association analysis

The genotype data of rs10795668 for cases and controls were shown in **Table 2**. The genotype distribution in controls complied with Hardy-Weinberg equilibrium (*P* = 0.433). Significant difference in genotype distribution was observed between cases and controls (*χ^2^* = 9.89, *P* = 0.007). In the unconditional Logistical Regression model, individuals with the AA genotype showed a significant decreased CRC risk compared with those carrying the GG genotype (OR = 0.51, 95%CI: 0.28–0.94). In the allelic model, A allele carriers also showed significantly decreased risk compared to those with the G allele carriers (OR = 0.70, 95%CI: 0.52–0.93). Additionally, significantly decreased risk of CRC was also found in additive model, with per-A-allele OR of 0.71 (95%CI: 0.54–0.94). Likewise, significant association between this polymorphism and decreased CRC risk was found in dominant model (OR = 0.65, 95% CI: 0.44–0.96). While no significant associations were found for AG versus GG and recessive model.

**Table 2:** Association between rs10795668 and colorectal cancer risk in a Chinese population.

	Case	Control				
Genotype	No. (%)	No. (%)	Crude OR (95%CI)^a^	*P* a	Adjusted OR (95%CI)^b^	*P* b
GG	230(48.9)	186(39.2)	1.00		1.00	
AG	187(39.8)	216(45.5)	0.70(0.53–0.92)	0.011	0.70(0.46–1.06)	0.093
AA	53(11.3)	73(15.3)	0.59(0.39–0.88)	0.010	0.51(0.28–0.94)	0.030
A vs. G			0.74(0.61–0.89)	0.002	0.70(0.52–0.93)	0.013
Additive model			0.75(0.62–0.90)	0.002	0.71(0.54–0.94)	0.017
Dominant model			0.67(0.52–0.87)	0.003	0.65(0.44–0.96)	0.030
Recessive model			0.70(0.48–1.02)	0.065	0.61(0.34–1.08)	0.088

a Crude results without adjustment.

b Adjusted by age, sex, smoking status, alcohol use and BMI.

#### Two-factor gene-environment interaction analyses

To explore the potential interactions between rs10795668 and smoking status, alcohol use and BMI, we performed two-factor gene-environment interaction analyses by unconditional Logistic Regression. The results were displayed in **Table 3**. No significant interaction was found according to the *P* value of the interaction term (*P*≥0.017). Among individuals with BMI≥24 kg/m^2^, carries with AA and AG genotypes showed significantly decreased risk of CRC compared with those with GG genotype, with an OR of 0.45 (95%CI: 0.22–0.91). However, the association was not found among individuals with BMI<24 kg/m^2^.

**Table 3:** Interaction analyses between rs10795668 and environment factors associated with CRC risk.

Variables	OR(95%CI)^a^	*P*
Total	0.65(0.44–0.96)	0.030^a^
**Smoking status**		0.902^b^
Never	0.63(0.40–1.00)	
Ever	0.59(0.28–1.24)	
**Alcohol use**		0.940^b^
Never	0.63(0.39–1.01)	
Ever	0.60(0.29–1.23)	
**BMI**		0.183^b^
<24 kg/m^2^	0.79(0.49–1.27)	
≥24 kg/m^2^	0.45(0.22–0.91)	

a OR of AA + AG vs. GG; derived from unconditional Logistic Regression after adjusting for age, sex, smoking status, alcohol use and BMI; *P*≤0.05 (significant).

b *P* value for the interaction term and the cutoff was 0.017(0.05/3) after the Bonferroni correction.

### Results of Meta-analysis

#### Characteristics of included studies

**Figure 1:**
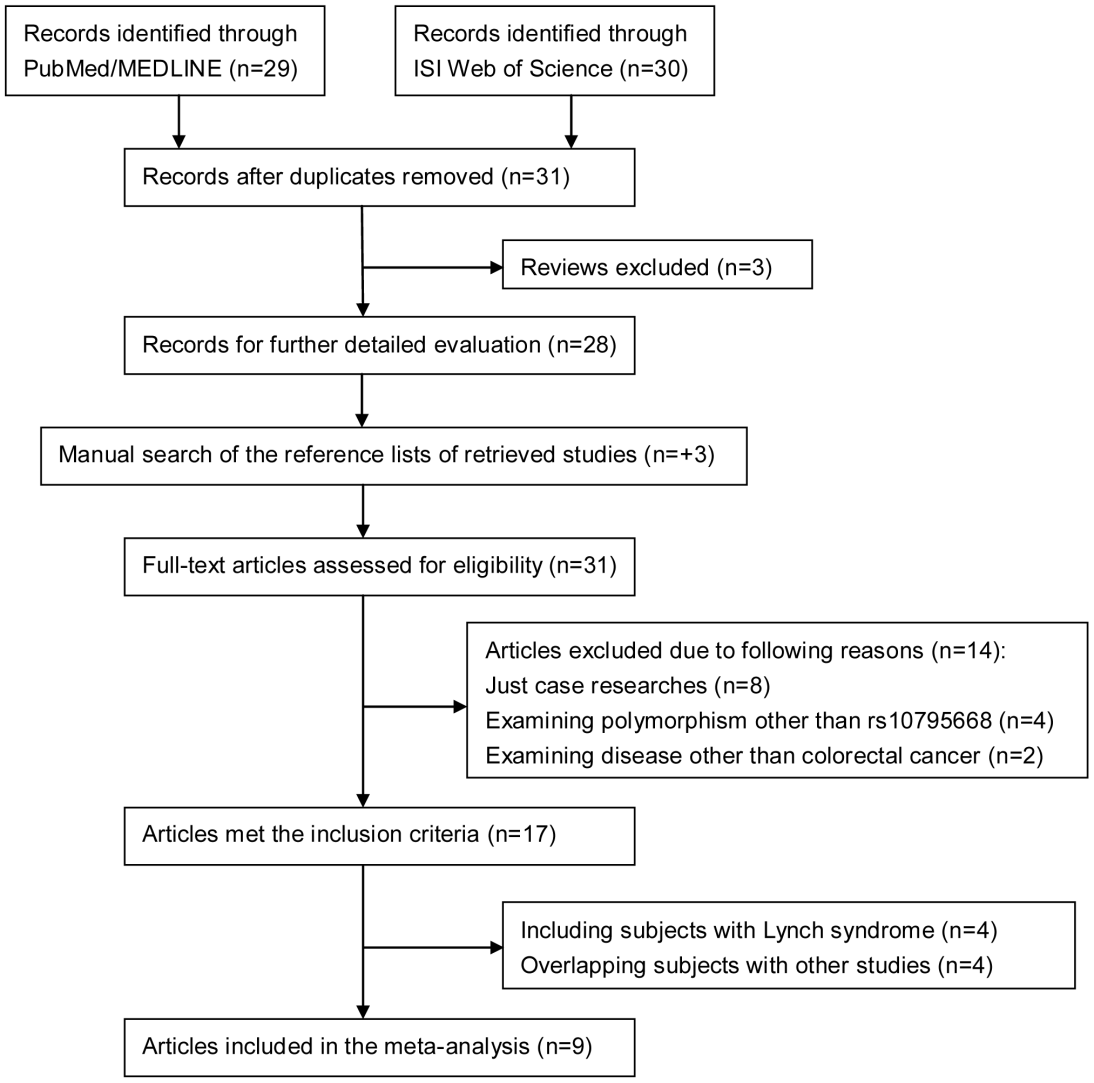
Flow chart of search strategy and study selection.

As shown in **Figure 1**, 31 potentially relevant articles were identified and screened, of which, 17 articles met the inclusion criteria. However, after further examination, we excluded 4 studies [26]–[28], [43] for including patients with Lynch syndrome and another 4 studies [44]–[47] for containing overlapping participants with the included studies. Finally, combining the current study, a total of 10 publications [6], [17], [18], [20]–[25] with 14 date sets comprising 39341 cases and 41566 controls were included in the meta-analysis (**Table 4**). Of these, 7 studies were conducted in Europeans, 5 in Asians and 2 in African Americans. Genotypes of rs10795668 in controls conformed to Hardy-Weinberg equilibrium for all included studies (*P*>0.01).

**Table 4:** Characteristics of studies on the association between rs10795668 polymorphism and colorectal cancer risk included in the meta-analysis.

First Author	Year	Country	Ethnicity	Study design	Study type	Genotyping method	Cases	Controls	Control source	HWE	MAF
Tomlinson IP	2008	Mixed	European	Mixed CC	GWAS	Illumina, KASPar	18831	18540	Mixed	Y	0.33
Kupfer SS (African Americans)	2009	America	African	CC	Replication	Sequenom MassARRAY	795	985	Population	Y	0.06
Kupfer SS (European Americans)	2009	America	European	CC	Replication	Sequenom MassARRAY	399	367	Population	Y	0.30
Von Holst S	2010	Sweden	European	Nested CC	Replication	DeCode test	1786	1749	Population	Y	0.34
Xiong F	2010	China	Asian	CC	Replication	PCR-RFLP	2124	2124	Population	Y	0.37
He J(European Americans)	2011	America	European	Mixed CC	Replication	TaqMan	1171	1534	Mixed	Y	0.31
He J(African Americans)	2011	America	African	Mixed CC	Replication	TaqMan	382	510	Mixed	Y	0.07
He J(Japanese Americans)	2011	America	Asian	Mixed CC	Replication	TaqMan	1042	1426	Mixed	Y	0.44
Ho JW	2011	HK, China	Asian	CC	Replication	Sequenom MassARRAY	892	890	Hospital	Y	0.38
Peters U (GWAS)	2012	Mixed	European	Mixed CC	GWAS	Illumina	2906	3416	Mixed	Y	0.32
Peters U (Replication)	2012	Mixed	European	Mixed CC	Replication	Sequenom MassARRAY, TaqMan	8161	9101	Population	Y	NA
Mates IN	2012	Romania	European	CC	Replication	DeCode test	153	182	Hospital	Y	0.30
Li FX	2012	China	Asian	CC	Replication	Sequenom MassARRAY	229	267	Hospital	Y	0.34
Current study	2012	China	Asian	CC	Replication	Sequenom MassARRAY	470	475	Population	Y	0.38

Abbreviation: HWE, Hardy-Weinberg equilibrium; MAF, minor allele frequency; CC, case-control study; GWAS, genome-wide association studies; NA, not available.

#### Frequency of A allele in control populations

No significant heterogeneity were observed in European and African American groups (*P_heterogeneity_* = 0.236 and 0.462, respectively). The pooled frequency using the fixed random-effects model were 0.327 (95%CI: 0.322–0.333) in European controls and 0.063 (95%CI: 0.051–0.075) in African American controls. There was significant heterogeneity among the Asian studies (*P_heterogeneity_*<0.001), and the pooled frequency was 0.385 (95%CI: 0.352–0.419) under random-effects model. These pooled frequencies were similar to those reported in dbSNP database of 0.353, 0.390 and 0.065 for Europeans, Asians and African Americans, respectively. Because of the different frequency distribution of the SNP rs10795668 across different populations, the meta-analysis was started in each ethnic group separately.

#### The meta-analysis of rs10795668 in associated with CRC

**Figure 2:**
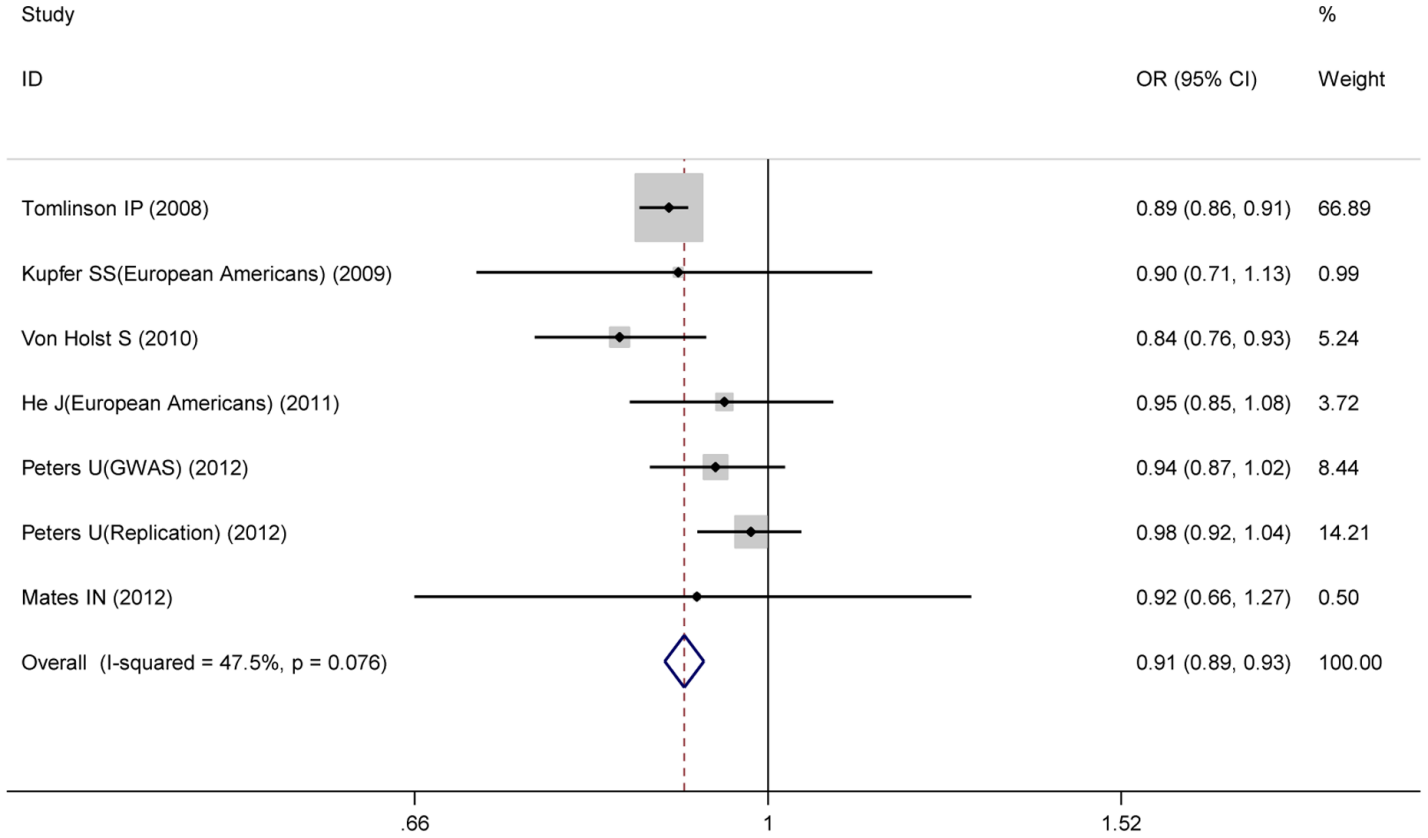
The forest plot of association of rs10795668 with colorectal cancer for European group.

**Figure 3:**
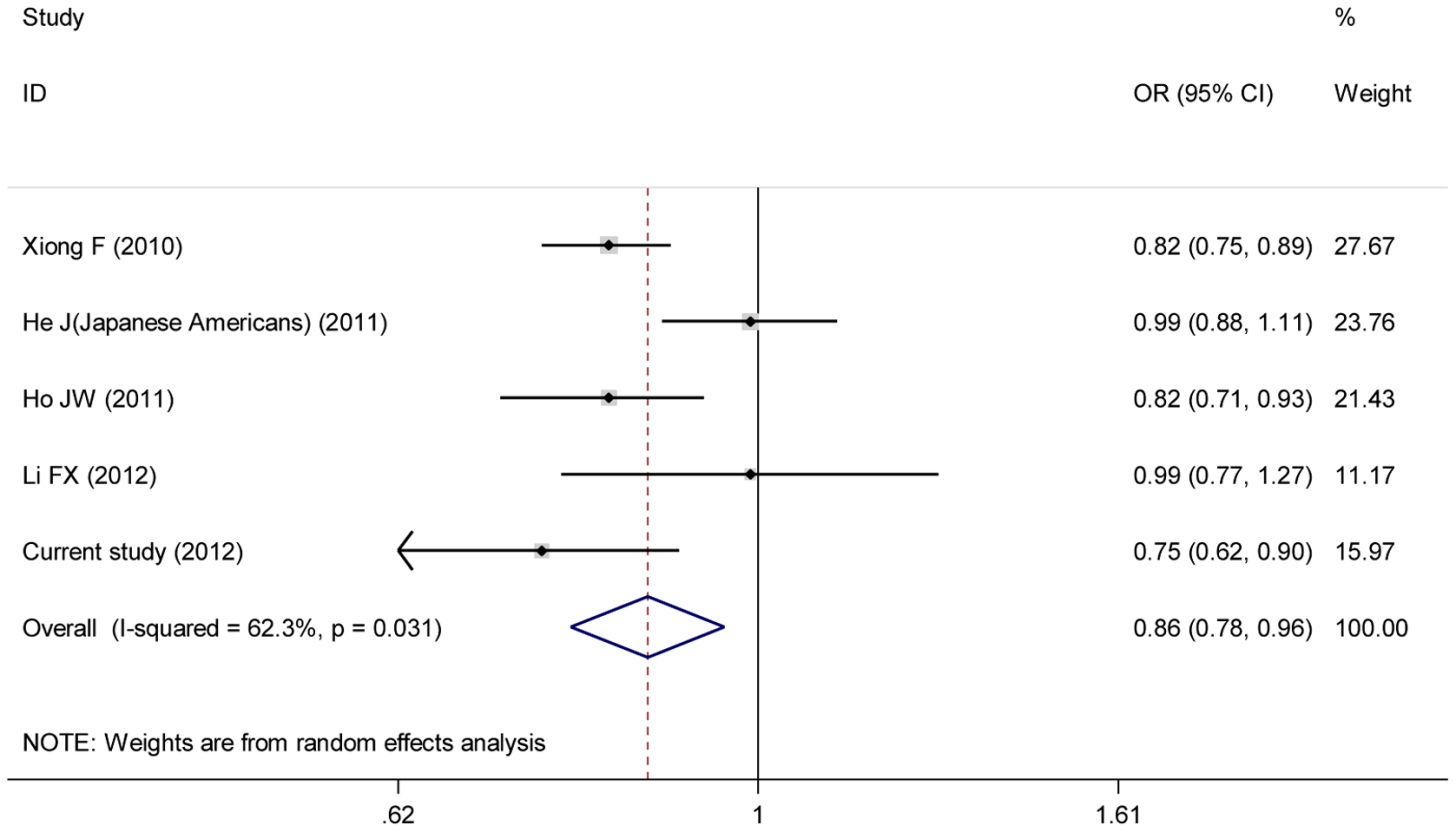
The forest plot of association of rs10795668 with colorectal cancer for Asian group.

**Figure 4:**
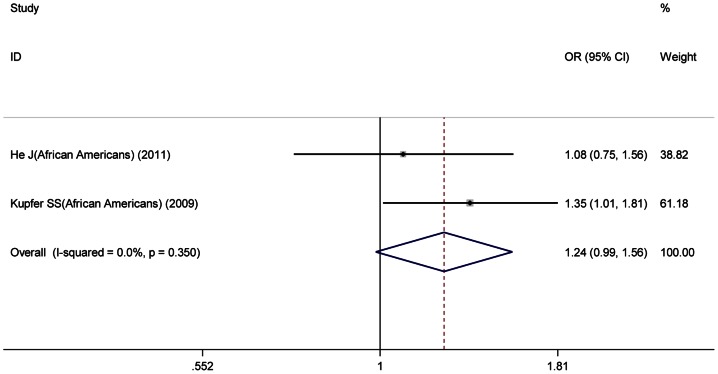
The forest plot of association of rs10795668 with colorectal cancer for African American group.

As shown in **Table 5**, only in Asian group significant evidence of heterogeneity was detected (*P_heterogeneity_* = 0.031, *I* ^2^ = 62.3%), therefore ORs in Asian group were pooled under random-effects model, and ORs in European and African American group were both pooled under fixed-effects model. Significant association between the variant and CRC risk was found both in European and Asian groups, with ORs of 0.91 (95%CI: 0.89–0.93) (**Figure 2**) and 0.86 (95%CI: 0.78–0.96) (**Figure 3**), respectively. While, in African American group, the variant showed borderline inverse association with CRC risk (OR = 1.24, 95%CI: 0.99–1.56) (**Figure 4**).

**Table 5:** Meta-analysis results of the rs10795668 in association with colorectal cancer risk.

Ethnicity	Data sets	Cases	Controls	Genetic model	OR(95%CI)^a^	*P* a	*Q* b	*P* b	*I^2^*	*T* c	*P* c
European	7	33407	34889	Additive model	0.91 (0.89–0.93)	1.85×10^−17^	11.42	0.076	47.5%	0.63	0.558
Asian	5	4757	5182	Additive model	0.86(0.78–0.96)	0.005	10.60	0.031	62.3%	0.24	0.823
African American	2	1177	1495	Additive model	1.24(0.99–1.56)	0.067	0.87	0.350	0.0%	-	-

a OR and *P* values were calculated in fixed or random effect model;

b Q and *P* values were calculated from Cochran's Q test;

c *t* and *P* values were calculated by Egger's test.

For the European data sets, additional analysis without the initial study [18] was performed, so as to avoid bias. After excluding the original study's samples, the association between the variant and CRC risk was still significant, with OR of 0.94(95%CI: 0.90–0.98, *P_heterogeneity_* = 0.242) (**Figure S1**).

#### Sensitivity analyses

To assess the influence of each individual study on the pooled additive OR, sensitivity analyses was performed by removing the individual study sequentially. As shown in **Figure S2**, the pooled ORs were similar before and after deletion of each study, indicating the robust stability of the current results.

#### Publication bias

Funnel plot and the Egger's test were used to assess publication bias. As reflected in **Figure S3**, the shape of the funnel plot seemed symmetrical. And the Egger's test did not detect any publication bias (**Table 5**).

## Discussion

The SNP rs10795668 located at 10p14 was first revealed to be associated with CRC risk by a multistage GWA study, but inconsistent results have been reported by following studies. The present case-control study found a significant association between rs10795668 polymorphism and CRC risk in Chinese population. Then, the following meta-analysis also suggested that the SNP was significantly associated with CRC risk. This meta-analysis was the first to integrate published GWA studies and replication studies to clarify the effect of rs10795668 variant on CRC risk.

The SNP rs10795668 maps to an 82-kb LD block (8.73–8.81 Mb) within 10p14 [18]. Tomlinson IP et al. investigated three additional SNPs in this LD block (rs706771, rs7898455 and rs827405) and found no evidence for more than one disease locus in the region[18]. Like most of risk variants identified by GWAS, rs10795668 resides outside the coding regions of genes. The nearest predicted genes are *BC031880*, located 0.4 Mb proximal to rs10795668, and *LOC389936*, located 0.7 Mb distally. Although the variant rs10795668 have been found to be associated with CRC risk, little is known about the function of the SNP. To investigate possible regulatory functions on the expression of neighboring genes, Loo LW conducted cis-expression quantitative trait loci analyses and found an significant association between the low colorectal cancer risk allele (A) for rs10795668 at 10p14 and increased expression of *ATP5C1* [48]. *ATP5C1* encodes the gamma subunit of the catalytic core (F1) of the mitochondrial ATP synthase, the enzyme complex responsible for ATP synthesis, known to play a central role in cellular respiration. A common event in tumor cells is the metabolic switch from respiration (in the mitochondria) to glycolysis (in the cytosol), often referred as “the Warburg effect” [49]. Multiple mechanisms may initiate this switch, one of which is a decrease in the expression of the beta subunit of ATP synthase (F1) (*ATP5B*), leading to the disruption of the catalytic function of the ATP synthase complex, an event that has been previously observed in multiple cancer types [50], [51]. The increased expression of *ATP5C1* associated with the A allele at rs10795668 would be consistent with maintaining the activities of ATP synthase and cellular respiration and potentially inhibiting tumor progression for colorectal cancer. However, *ATP5C1*, located at10p15.1, is not the closest neighboring gene. Some studies [52], [53] suggest that the GWAS risk variants may not preferentially regulate genes that are closest. The transcriptional regulatory mechanisms impacted by allelic status may involve complex chromatin confirmation states and function within a tissue specific context. Besides, Most of variants identified by GWAS imply the probability of being in linkage with the “real” causal variants[54], so it is possible that the polymorphism is in linkage disequilibrium with “real” causal loci. What's more, understanding the biological function of these loci, focusing on non-coding variants, perhaps is the greatest challenge in the ‘post-GWAS’ era[55].

The Meta-analysis results showed significant association between the variant rs10795668 and CRC risk both in European and Asian population, but in African Americans the association was borderline significant and in the opposite direction. The pooled A allele frequency across different ethnic population varied. Besides, it was noted that Kupfer SS et al. also found that there was genetic heterogeneity in CRC associations in Americans of African versus European descent[24]. The sensitivity analyses and publication bias assessment indicated the current results from this meta-analysis were reliable.

Some limitations in the case-control study and the meta-analysis need to be addressed. First, the sample size of our case-control study was relatively small, resulting in the relatively inadequate statistical power. Second, in the meta-analysis, the number of published studies was still insufficient for the analyses in Asian and African American group, which might mask or exaggerate the possible true association. Third, although significant association between rs10795668 and CRC has been confirmed in this study, the functional experiments have not been done, so whether this variant is protecting remained uncertain.

In conclusion, our case–control study in a Chinese population and the meta-analysis verified that the variant rs10795668 was significantly associated with CRC risk. Further functional verification should be conducted to confirm the findings, which may help unravel the underlying mechanisms of this variant in CRC development and progression.

## Supporting Information

Figure S1 **The forest plot of association of rs10795668 with colorectal cancer for European group without the original study. (TIF)**
(TIF)

Figure S2 **Sensitivity analyses for European group. (TIF)**
(TIF)

Figure S3 **Funnel plot of publication bias for European group. (TIF)**
(TIF)

Checklist S1 **The PRISMA 2009 checklist.**
(DOC)
