# Supplementary figures and images for: The Genetic Variant on Chromosome 10p14 Is Associated with Risk of Colorectal Cancer: Results from a Case-Control Study and a Meta-Analysis
Source: PLoS One. 2013 May 22;8(5):e64310. doi: 10.1371/journal.pone.0064310 (PMC3661459; doi:10.1371/journal.pone.0064310)

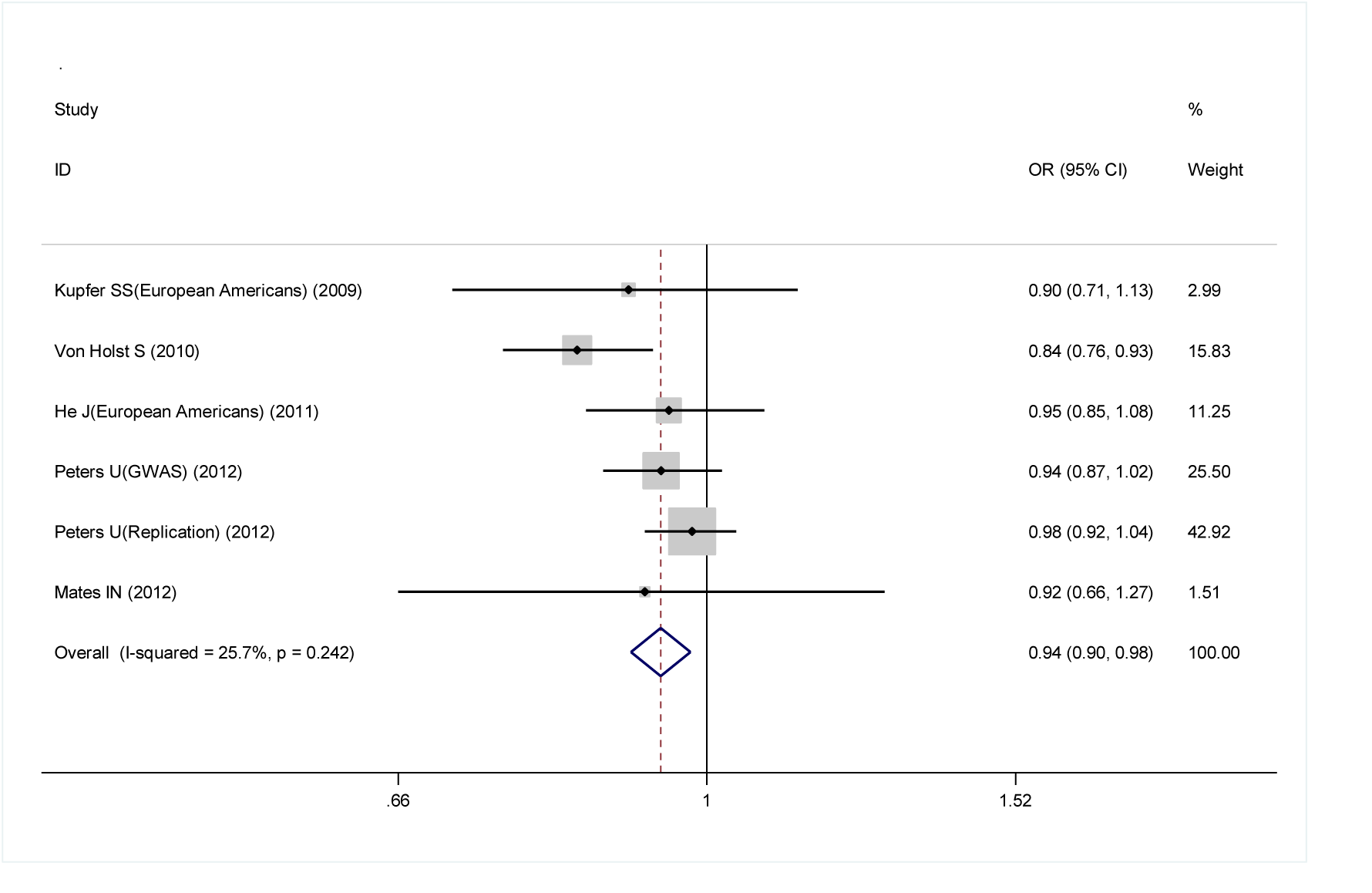

Supplement: Figure S1 — The forest plot of association of rs10795668 with colorectal cancer for European group without the original study. (TIF) (TIF) [file pone.0064310.s001.tif]
